# Sustainable and Ecofriendly Approach of Managing Soil Born Bacterium *Ralstonia solanacearum* (Smith) Using Dried Powder of *Conyza canadensis*

**DOI:** 10.3390/pathogens9050327

**Published:** 2020-04-27

**Authors:** Ke Chen, Raja Asad Ali Khan, Wen Cao, Meng Ling

**Affiliations:** 1 School of Life Science and Engineering, Southwest University of Science and Technology, Mianyang 621010, Sichuan, China; caowen@swust.edu.cn (W.C.); lingm0808@163.com (M.L.); 2 Institute of Vegetables and Flowers, Chinese Academy of Agricultural Sciences, Beijing 100081, China

**Keywords:** environment friendly, IDM, soil amendment, phytopathogenic bacteria, bacterial wilt

## Abstract

Bacterial wilt disease caused by *Ralstonia solanacearum* is a devastating plant disease that inflicts heavy losses to the large number of economic host plants it infects. The potential of dried powder of the *Conyza canadensis* to control bacterial wilt (BW) of tomato was explored in vitro and in planta. Three application times (16 days before transplanting (DBT), 8 DBT and 0 DBT), three plastic-mulch durations (10 days plastic mulching (DPM), 5DPM and 0DPM) and four doses viz. 0 g, 8 g, 16 g and 24 g of the plant powder were evaluated. SEM analysis was also conducted to observe the change in bacterial cell morphology. Ethanol extract of dried *C. canadensis* in different concentrations inhibited the in vitro growth of *R. solanacearum* by as much as 98% of that produced by ampicillin. As evident from the scanning electron micrograph, the highest concentration produced severe morphologic changes, such as rupture of the bacterial cell walls and cell contents leaked out. Results from application time and dose experiment demonstrated that the highest powder dose viz. 24 g kg^−1^ mixed with infested soil 16 DBT gave maximum root length (34.0 ± 2.5 cm), plant height (74.3 ± 4.7 cm), fresh biomass (58.3 ± 4.3 g), reduction in bacterial population (1.52 log10) and resulted in lowest AUDPC value (1156.6). In case of mulching duration and dose experiment the maximum root length (39.6 ± 3.2 cm), plant height (78.3 ± 5.8 cm), fresh biomass (65.6 ± 4.9 g) reduction in bacterial population (1.59 log10) and lowest AUDPC value (1251.6) was achieved through the application of highest powder dose viz. 24 g kg^−1^ and longest plastic mulching duration of 10 DPM. The better results of highest dose and longer application time can be explained on the basis of higher amounts of anti-microbial plant bio-active compounds in highest dose and the longer exposure time of the pathogen to these chemicals. The better results of longer mulching duration are due to faster and more complete decomposition (because of 10-days-long plastic-mulch-provided increased solar heat) of the dried powder which produced more amounts of volatile and non-volatile bactericidal compounds. Our results clearly suggest that the use of 24 g kg^−1^ dried plant powder of *C. canadensis* plastic-mulched for two weeks could be used as a reliable component of the integrated disease management program against BW.

## 1. Introduction

Tomato is one of the most consumed vegetables, grown worldwide in the field or greenhouse and has potential health benefits [1]. It is significant cash crop for medium-scale and small land holders. Tomatoes are recognized as healthy food and considered as important source of vitamins. Therefore, cultivation acreage, production, and consumption of tomatoes were substantially increased in worldwide [2]. Due to perennial growth on a large scale; tomato is susceptible to various biotic and abiotic factors which adversely affect the plant growth, crop yield, different biochemical changes and physiological processes in plants [3]. Among biotic factors the bacterial diseases are one of the major constraints in tomato production. *Ralstonia solanacearum* a Gram-negative bacterium, considered to be one of the most devastating plant pathogens having global distribution and significant economic impact worldwide [4]. It causes bacterial wilt disease in many plants and is particularly devastating to tomato [5,6], leading to substantial losses in tropical and other regions with warm temperature. It has a huge genetic diversity which affects tropical, subtropical, and warm temperate region [7]. 

*Ralstonia solanacearum* can survive in plant debris, infected plants host weeds and spread from one field to another by irrigation or flood water, soil, farm equipment, workers and weeds which usually grow along waterways and it is difficult to manage due to complication in biology, nature of infestation and wide host range [8]. Its management involves the use of resistant varieties and chemical control [9,10]. However, the use of resistant varieties has not always been successful and shows a negative correlation between resistance and yield [11]. In addition, the resistance of these varieties is often strain-specific, there were reports of bacteria developing resistance to the chemicals [12,13]. Therefore, integrated approach combining host plant resistance, cultural and biologic control measures seems effective. Although excellent attempts were made in management of R. solanacearum, still there is great opportunity to contribute to this problem for a stable solution. Varied chemical, cultural, agronomical, biologic, biotechnological and biochemical approaches, etc. were used in addressing problem of Ralstonia with different levels of success [14].

Soil amendment with organic matter having antimicrobial activity is one of the potential approaches for the management of soil borne pathogens. This technique also improves the chemical, biologic and physical properties of soil and regulates the growth and development of the plant. After degradation in the soil, encapsulated antibacterial organic matter releases some natural chemicals that have various inhibitory properties [15]. This technique was used successfully for the control of *R. solanacearum* both in vivo and in vitro. Soil amendment with dried powder or green manure of different medicinal plants or weeds such as *Withania coagulans*, *W. somnifera*, *Xanthium strumarium* resulted in significant reduction in bacterial wilt disease in tomato plants [16,17,18]. Their aqueous extracts effectively inhibited the growth of *R. solanacearum* which indicates the presence of antibacterial compounds in these plants. In this study we used the dried powder of *Conyza canadensis* for the management of bacterial wilt disease in tomato. *C. canadensis* (syn. *Erigeron canadensis* L.), known as ‘Canadian fleabane’ or ‘horseweed’, is native throughout of North America and is also widespread in Europe and Asia. It is an annual plant, erecting with one to several sparse hairy stems reaching 10–180 cm high [19]. Several studies reported the antibacterial, antifungal and antiviral potential against human pathogen [20,21,22,23,24,25], however its potential to control plant pathogens and especially plant pathogenic bacteria has not been evaluated yet. The aim of this work was to evaluate the antibacterial potential of *C. canadensis* in vitro and the study also was extended to investigate the effect of different dried powder doses of *C. canadensis* against bacterial wilt disease in tomato under greenhouse conditions. 

## 2. Results

### 2.1. In Vitro Antibacterial Evaluation

**Figure 1:**
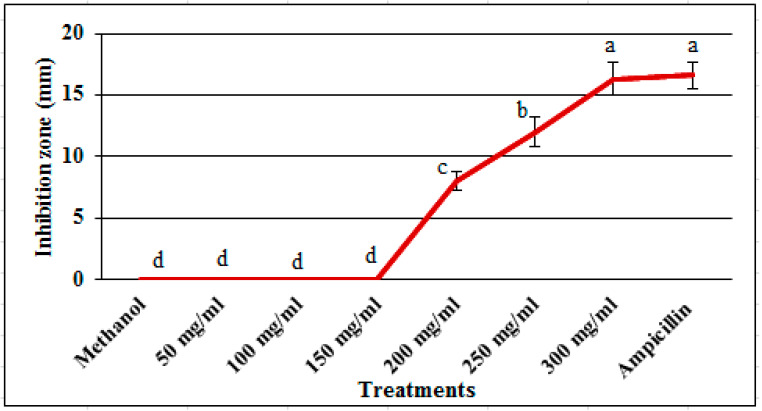
In vitro growth inhibition of *R. solanacearum* by different concentrations of ethanol extracts of *Conyza canadensis*. Values represent the means of ten observations (n = 10). Same letters show no significant (*P* < 0.05) difference among the treatments. Bars represent the standard deviation.

**Figure 2:**
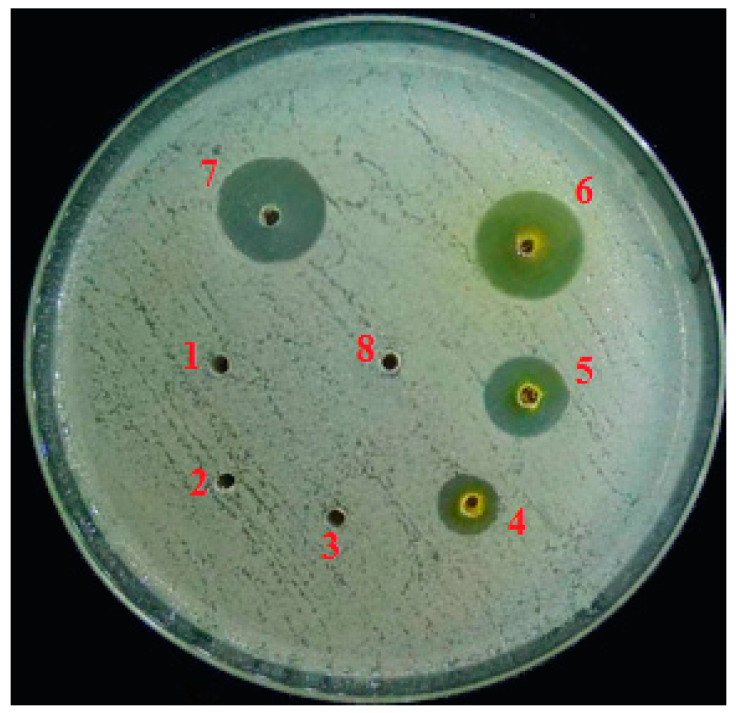
Growth of inhibition zones produced by ethanol extracts of *Conyza canadensis* against *Ralstonia solanacearum*. 1: 50 mg/mL, 2: 100 mg/mL. 3: 150 mg/mL. 4: 200 mg/mL, 5: 250 mg/mL, 6: 300 mg/mL, 7: Ampicillin, 8: Methanol.

Ethanol extracts of *C. canadensis* in different concentrations showed significantly different growth inhibition of R. solanacearum (Figure 1). The first three concentrations i.e., 50, 100 and 150 mg/mL did not show activity while the concentrations 200, 250 and 300 mg/mL showed increasingly higher inhibition zones. The highest concentration 300 mg/mL produced inhibition zone of 16.3 ± 1.6 mm that was similar to inhibition zone (16.6 ± 1.2 mm) produced by positive control streptomycin. Negative control treatment methanol showed no activity (Figure 2).

### 2.2. Morphologic Observation of Bacterial Cells

**Figure 3:**
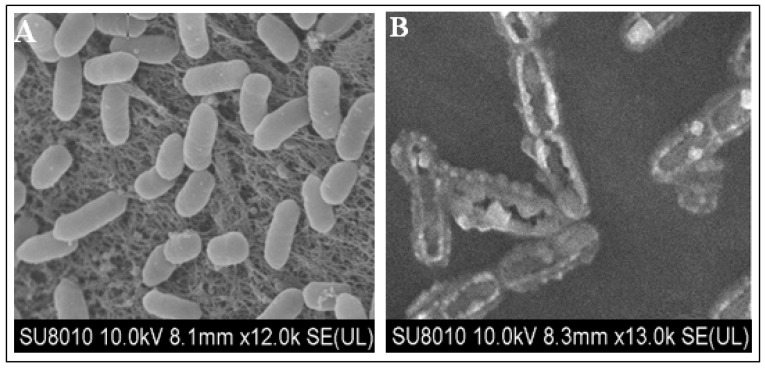
SEM micrographs of *Ralstonia solanacearum* cells. (**A**) Untreated healthy cells, (**B**) Treated bacterial cells with *Conyza canadensis* extracts at 300 mg/mL.

Scanning electron microscopy (SEM) analysis of treated and untreated bacterial cells showed clear morphologic changes in bacterial cells. Micrographs of bacterial cells treated with ethanol extracts of *C. canadensis* exhibited severe cell disruptions (Figure 3B). As compared to control cells, the cell membrane and cell wall of treated cells were broken; cell shape was deformed and degraded while untreated bacterial cells showed normal cell morphology without any disruption (Figure 3A).

### 2.3. In Planta Antibacterial Evaluation

#### 2.3.1. Experiment 1: Application Time and Dose Effect

**Figure 4:**
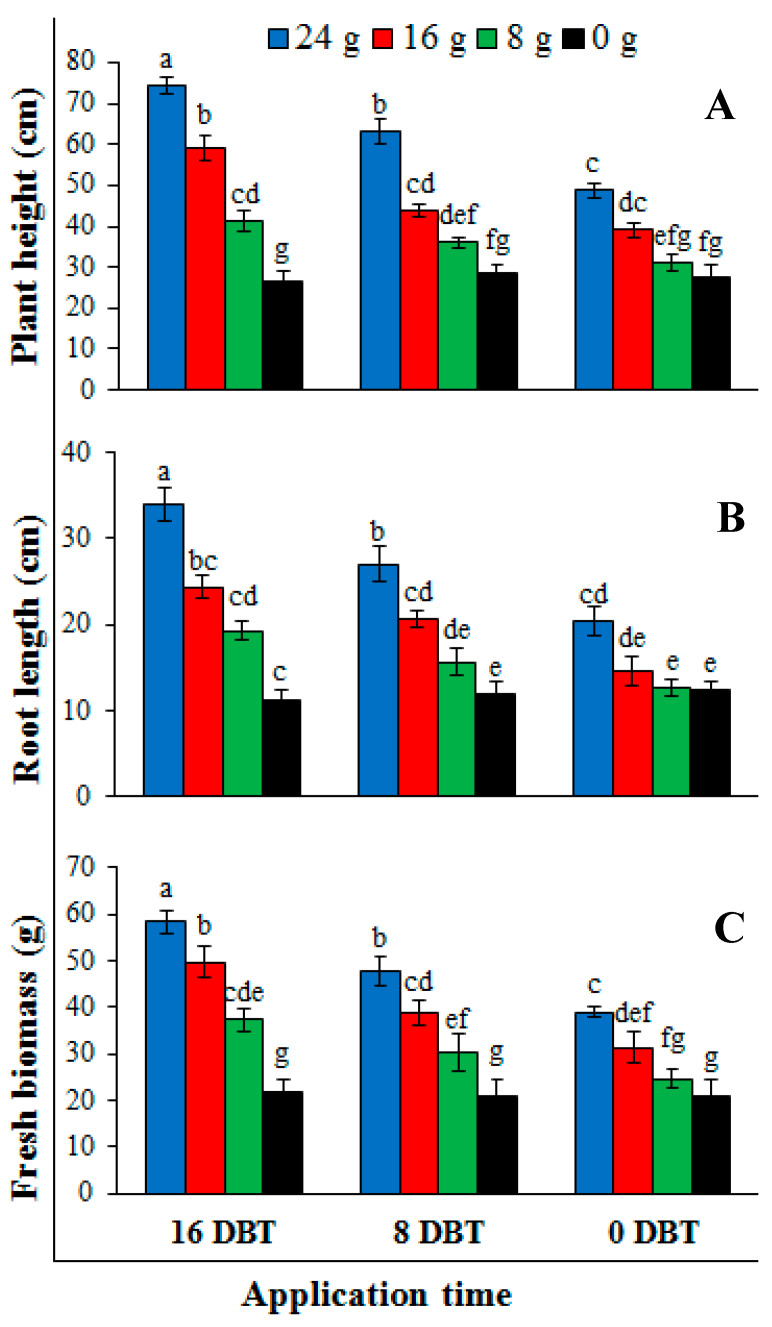
Effect of *Conyza canadensis* dried powder doses (0 g, 8 g, 16 g and 24 g) and application time (0 DBT, 8 DBT and 16 DBT) applied to artificially inoculated soil with *Ralstonia solanacearum* on (**A**) plant height, (**B**) root length and (**C**) fresh biomass of tomato plants. Each value is a mean ± SD of 8 replicates. DBT: Days before transplanting. Mean values showing different lettering are significantly different (*P* ≤ 0.05) as per Fisher’s protected LSD test. Bars represent standard deviation.

**Figure 5:**
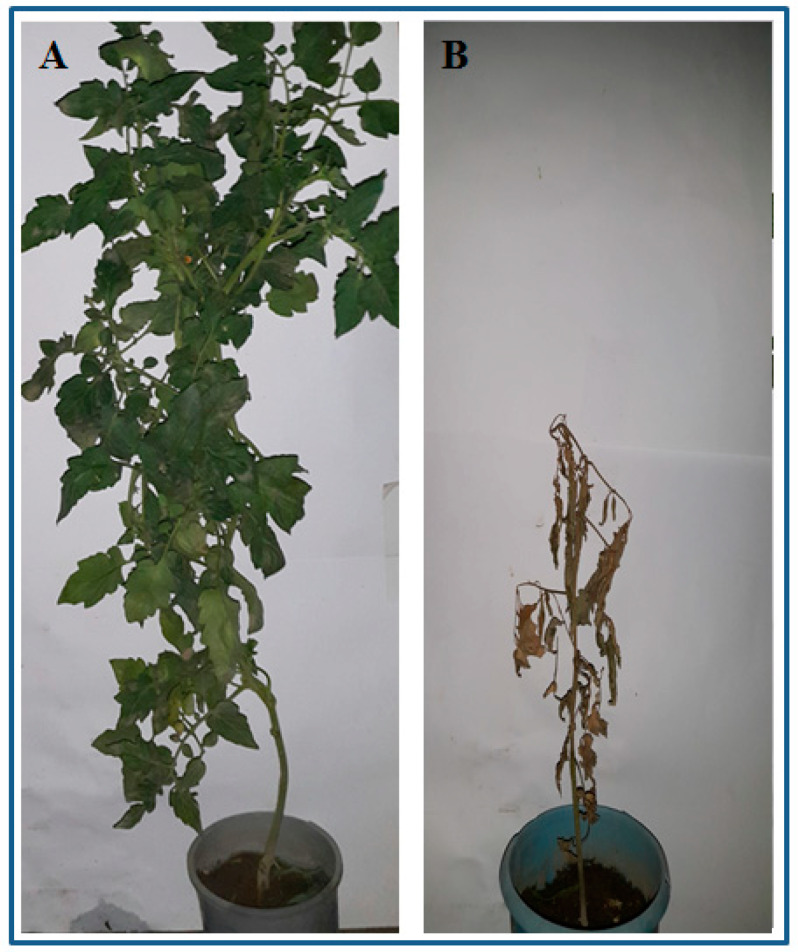
(**A**) Effect of dried plant powder at 24 g kg^−1^ inoculated soil applied 16 days before transplanting on growth of tomato plant and (**B**) Control (unamended soil).

Results on plant parameters showed that different application times and doses of dried plant powder significantly affected the plant height, root length and fresh biomass of tomato plants transplanted in artificially inoculated soil with *R. solanacearum* (Figure 4). It was noticed that increase in application time and dose of dried plant powder resulted in enhancing the plant height, root length and fresh biomass. Maximum plant height (74.3 ± 4.7 cm), root length (34.0 ± 2.5 cm) and fresh biomass (58.3 ± 4.3 g) were achieved by the highest dose (24 g) of the plant powder applying at 16 DBT (Figure 5) followed by 16 g of dried plant powder applying at 8 DBT while after control (un-amended soil; 0 g) the lowest dose (10 g) of dried plant powder gave the minimum values for plant height, root length and fresh biomass of tomato plants 

#### 2.3.2. Experiment 2: Plastic Mulch Duration and Dose Effect

**Figure 6:**
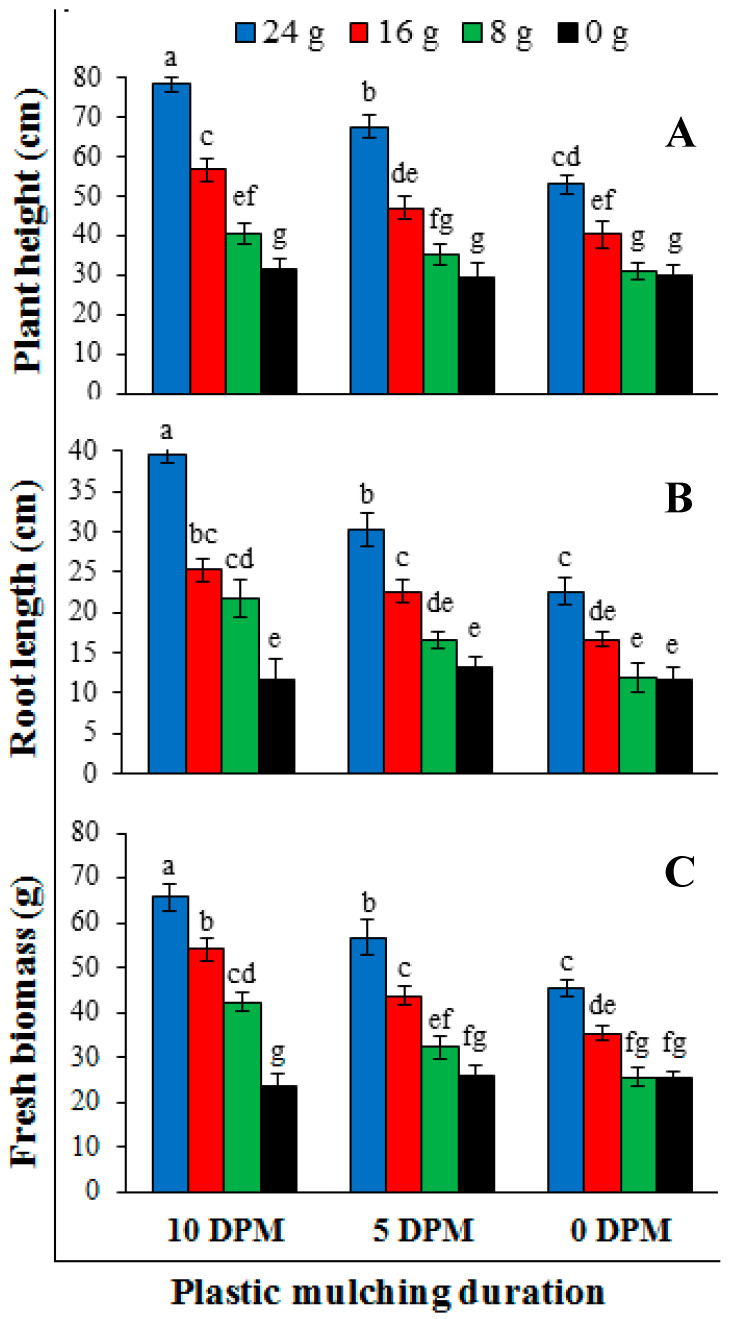
Effect of *Conyza canadensis* dried powder doses (0 g, 8 g, 16 g and 24 g) and plastic mulching duration (0 DPM. 5 DPM and 10 DPM) applied to artificially inoculated soil with *Ralstonia solanacearum* on (**A**) plant height, (**B**) root length and (**C**) fresh biomass of tomato plants. Each value is a mean ± SD of 8 replicates. DPM: Days plastic mulching. Mean values showing different lettering are significantly different (*P* ≤ 0.05) as per Fisher’s protected LSD test. Bars represent standard deviation.

**Figure 7:**
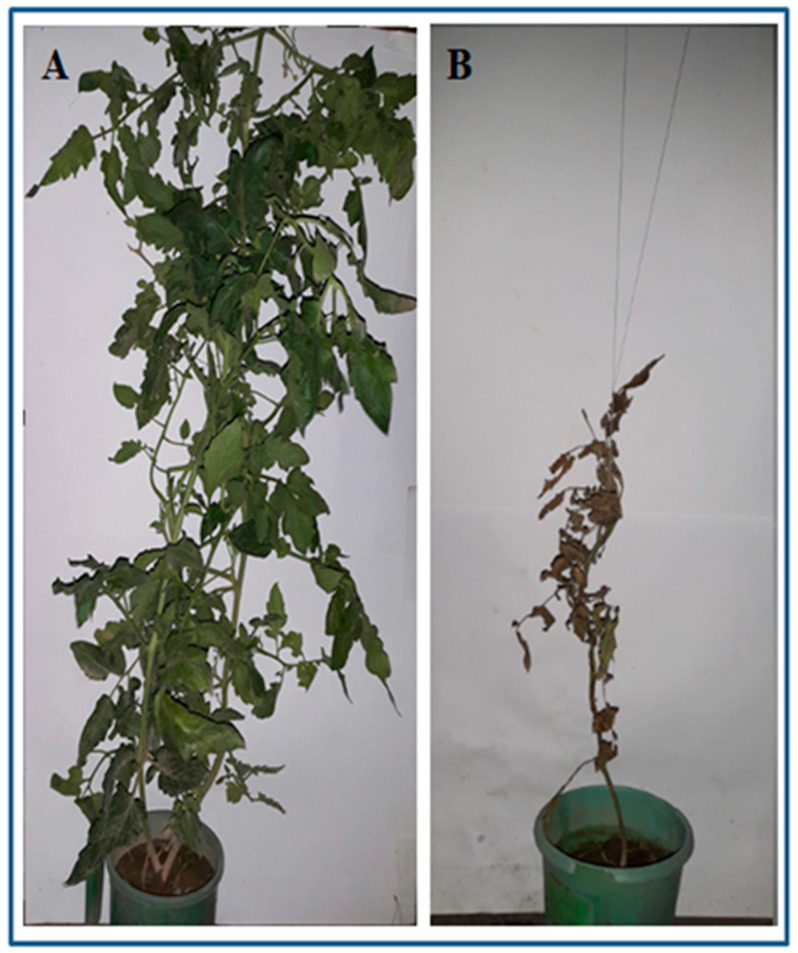
(**A**) Effect of dried plant powder at 24 g kg^−1^ inoculated soil and plastic mulched for 10 days on growth of tomato plant and (**B**) Control (un-amended soil).

Plant growth parameters significantly affected by the application of different duration of plastic mulching and doses of dried plant powder. Highest dose of the dried plant powder and plastic mulching for longer duration effectively increased the plant growth parameters (Figure 6). The combination of 24 g dried plant powder and plastic mulching for 10 days gave maximum plant height (78.3 ± 5.8 cm), root length (39.6 ± 3.2 cm) and fresh biomass (65.6 ± 4.9 g) (Figure 7). The application of 16 g dried powder and 5 days plastic mulching was ranked as second-best treatment combination while 8 g dried plant powder gave the lowest values for plant growth parameters after control (0 g).

### 2.4. Effect of Plant Powder, Application Time and Plastic Mulching on Soil Population of R. solanacearum

**Figure 8:**
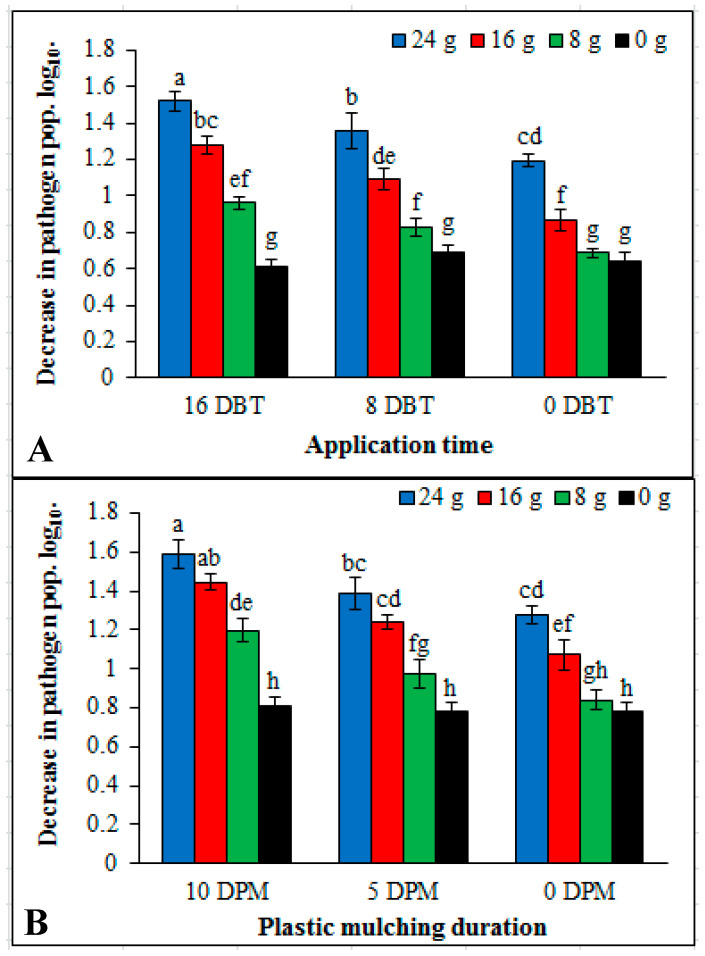
Effect of dried powder doses (0 g, 8 g, 16 g and 24 g), application time of *Conyza canadensis* and plastic mulching duration on decrease in soil pathogen population. (**A**) Application time and dose effect, (**B**) plastic mulching duration and dose effect. Each value is an average cfu/g dry soil (initial log_10_-final log_10_). DPM: days plastic mulching. DBT: days before transplantation. Mean values showing same lettering are not significantly different (*P* ≤ 0.05) as per Fisher’s protected LSD test. Bars represent standard deviation.

Different doses of dried plant powder, application timings and plastic mulching duration significantly reduced the pathogen population in the soil. Increase in reduction of bacterial population was achieved by increasing the dose of dried plant powder, application time and mulching duration. In case of application time and dose effect the highest reduction (1.52 log_10_) was achieved by the application of 24 g dried plant powder applied 16 DBT while for plastic mulching duration effect the 24 g dried plant powder and 10 days plastic mulching (DPM) gave highest reduction (1.59 log_10_) in soil population of pathogen than other mulching durations. This treatment was followed by 16 g dried plant powder applied at 8 DBT and 5 DPM. The lowest reduction (0.64 log_10_) and (0.78 log_10_) were noted with 0 g, 0 DBT and 0 g, 0 DPM treatment combinations respectively (Figure 8).

### 2.5. Effect of Plant Powder, Application Time and Plastic Mulching on AUDPC

**Figure 9:**
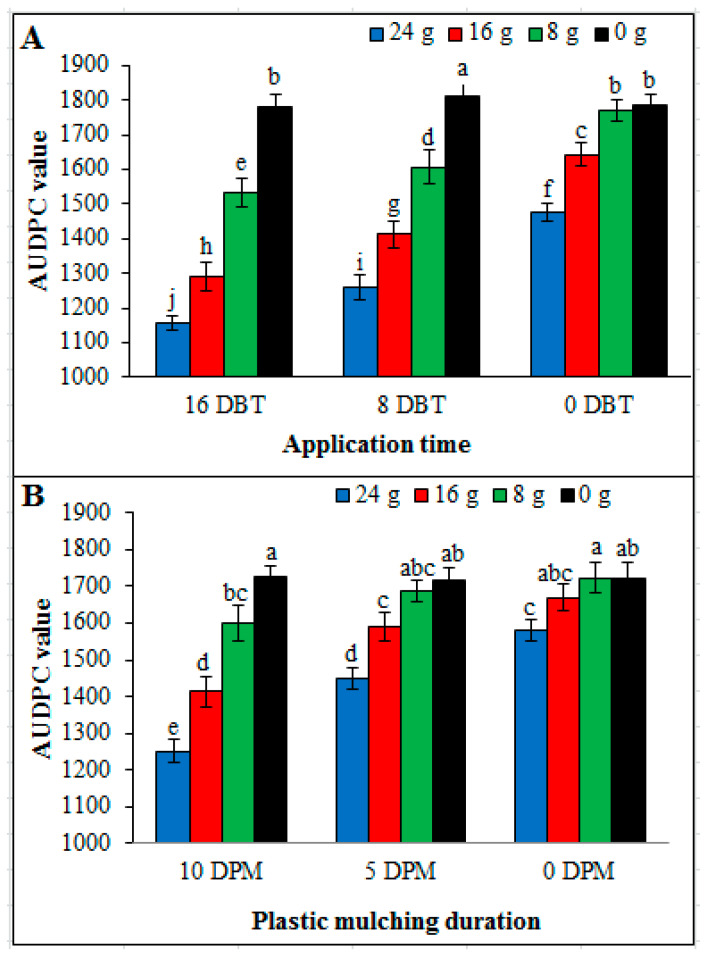
Effect of dried powder doses (0 g, 8 g, 16 g and 24 g), application time of *Conyza canadensis* and plastic mulching duration on AUDPC. (**A**) Application time and dose effect, (**B**) Plastic mulching duration and dose effect. DPM: days plastic mulching. DBT: days before transplantation. Mean values showing same lettering are not significantly different (*P* ≤ 0.05) as per Fisher’s protected LSD test. Bars represent standard deviation.

Disease progress over time was quantified by determining AUDPC. As obvious from the results, AUDPC values were significantly affected by different doses of dried powder applied at different timings and different duration of plastic mulching. The smallest AUDPC value was obtained by using 24 g dried powder applied at 16 DBT which was followed by 16 g and 8 DBT. In case of plastic mulching duration and doses, longest plastic mulch duration 10 DPM and highest dose 24 g dried powder gave minimum AUDPC value. Control (un-amended soil) treatments i.e., 0 g dried powder, 0 DBT and 0 DPM exhibited highest AUDPC values (Figure 9).

### 2.6. Comparison of Application Time (16 DBT) without Plastic Mulching and 10 Days Plastic Mulching Effect

**Figure 10:**
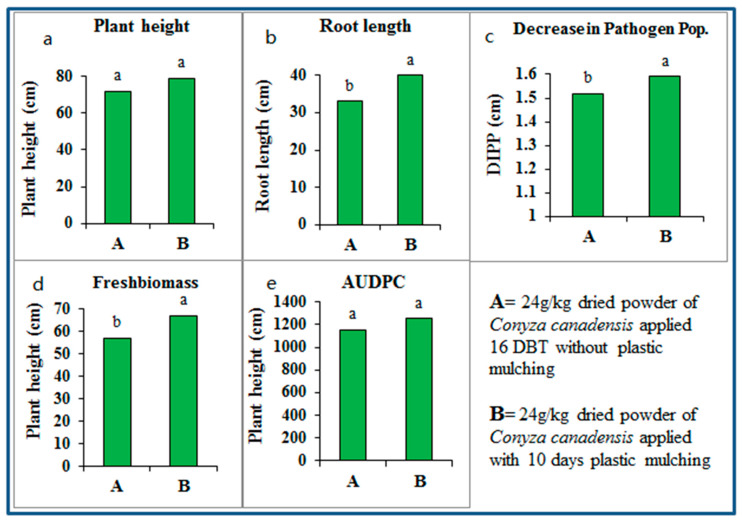
Comparison of *Conyza canadensis* dried powder at 24 g/kg soil, applied 16 days before transplantation (16 DBT) without plastic mulching and with 10 days plastic mulching (10 DPM). Mean values showing same lettering are not significantly different (*P* ≤ 0.05) as per Fisher’s protected LSD test. (**a**) Plant height; (**b**) Root length; (**c**) Decrease in Pathogen Pop.; (**d**) Freshbiomass and (**e**) AUDPC.

To compare the effect of *Conyza canadensis* dried powder with or without plastic mulching the results of the best treatments i.e., 24 g/kg from two experiments were compared through one-way ANOVA following LSD test. No significant difference was observed in plant height and AUDPC while application of 24 g/kg *Conyza canadensis* dried powder with ten days plastic mulching significantly enhanced the root length, fresh biomass and caused more decrease in soil pathogen population than application of 24 g/kg *C. canadensis* dried powder applied at 16 DBT without plastic mulching in Figure 10.

## 3. Discussion

Utilization of plant-based materials—being an environment-friendly, easily biodegradable and relatively non-phytotoxic technique—is a potentially effective and attractive component of integrated disease management (IDM) against bacterial wilt [26,27,28]. Results of this study showed strong antibacterial activity of methanol extract of *C. canadensis* against bacterial wilt pathogen *R. solanacearum* which suggested the potential use of its powder for the management of bacterial wilt disease in tomato plants. It was noticed that antibacterial potential of *C. canadensis* extract was dose dependent. The antibacterial activity could be attributed to different antimicrobial secondary metabolites produced by *C. canadensis*. A number of phytochemical studies reported that *C. canadensis* contains several bioactive secondary metabolites having antibacterial, antifungal and antiviral activities [20,21,22,23,24,25]. Plant bio-active secondary metabolites damage bacterial cells by different mechanisms including membranes disruption [29,30,31,32,33,34,35]. Rupturing of cell membranes of *R. solanacearum* cells treated with *C. canadensis* extracts was observed in SEM analysis which supports our *in vitro* studies. These findings are in line with previously reported results by different researchers. Cell wall of *Pseudomonas aeruginosa* and *Staphylococcus aureus* was found broken and cells appear shrunken when treated with methanol extracts of *Ocimum basilicum* L and observed in SEM. In another study reported ruptured and misshapen cell walls of *Staphylococcus epidermidis* by aqueous extracts of *Aquilaria crassna* was reported [36]. 

Along with other factors, the effectiveness of a plant product to control a plant disease also depends upon the amounts, time and method of application of the plant to be used as organic amendment. Results of our in-planta studies indicated that soil mixing of dried powder of *C. canadensis* at different rates and applied at different times significantly suppressed BW, declined the pathogen count in soil and enhanced the plant growth characters. Application of higher dose (24 g kg^−1^ soil) of dried powder applied 16 DBT was found to be superior to the lower doses applied fewer days before transplanting. Higher amount of the dried powder obviously has more antimicrobial compounds and longer exposure time helps in more thorough decomposition of the organic amendment, releasing more plant bio-active compounds. Moreover, the *R. solanacearum* present in the infested soil had more exposure to the toxic substances in case of 16 DBT than in 8 DBT. This decreased the pathogen populations in soil which resulted in less disease and enhanced plant growth characters. Other researchers also reported similar results for the management of bacterial wilt in tomato as well as other plant disease. For example, using dried powder (40 g kg^−1^ potted soil) of *A. vasica* and *Calotropis procera* suppressed BW significantly and enhanced plant growth and yield in tomato plants [37]. Mulching of flowering-stage of Brassica species released thiocyanates, isothiocyanates and nitriles which infected population of *R. solanacearum* in soil [38]. Likewise, release a volatile compound thymol in soil by decomposition of Thymus plants was effectively managed BW of tomato [39]. Dried powders [37,40,41,42] as soil organic amendments prepared from plants with bio-active compounds have been demonstrated to be effective against different plant diseases. A correlation was found between the decrease in nematode population in tomato roots and increasing plant material doses [41]. Higher doses of green manures release more anti-microbial chemicals, and therefore achieve greater control of plant diseases.

Release of bio-active compounds by decomposition of applied plant powder in soil may either act as elicitors to induce systemic acquired resistance (SAR) in host plants or damage the pathogens directly [42,43]. Plants having activated SAR can protect themselves better against pathogens which resulted in reduced disease levels [44,45]. Aqueous extracts of *Punica granatum*, *Eucalyptus globulus* and *Hibiscus sabdariffa*, were found to be able to induce SAR against bacterial wilt in potato plants [46].

In case of plastic mulching effect, the application of plastic mulching (10 DPM) significantly increased plant growth and decreased disease severity than without plastic mulching (0 DPM). Our results of reduction in disease severity through longer duration of plastic mulching i.e., for 10 days could be explained on the basis of quicker decomposition of *C. canadensis* powder under increased solar heat (because of plastic mulching) and quicker and more release of anti-microbial compounds. This practice decreased the soil populations of *R. solanacearum* because bacterium is heat-sensitive [47]. It is killed when wet soil temperatures reach 45 °C or above for 2 days. Furthermore, plastic mulching could trap the volatile compounds released by the fast-decomposing dried powder under high temperature conditions [48]. This further decreases the soil populations of the bacterium which results in low disease severity of BW. Many areas of China which grow tomatoes commercially are hot during later part of the summer and spring. Therefore, through plastic mulching the target temperature of 45 °C could be easily achieved. Taking the advantage of cheap local labor, the dried powder could be applied to individual tomato plants in a target-application manner for reducing the in-put costs. Comparison of the effect of highest dose 24 g/kg applied 16 DBT without plastic mulching and with 10 days plastic mulching revealed that 10DPM was superior in terms of suppressing soil pathogen population, root length and plant fresh biomass. Though the other two parameters i.e., AUDPC and plant height were not affected by plastic mulching application but still plastic mulching can be considered as effective treatment because of taking less time i.e., 10 DPM than 16 DBT without plastic mulching. Hence, through the application of plastic mulching we can manage the bacterial wilt disease more effectively in less time (10 days) than 16 DBT without mulching. To sum up, the results of this study suggest that the mixing of dried *C. canadensis* powder into the soil could act as ecofriendly, low-cost and effective disease management tool for BW in tomato grown in pots and green houses. Obviously, it will also be helpful in field and possibly for other crops which should be evaluated for conformation. 

## 4. Materials and Methods

### 4.1. Bacterial Culture and Preparation of Inoculum

*R. solanacaerum* was obtained from the phyto-pathogenic bacterial culture collection of the School of Life Science and Engineering, Southwest University of Science and Technology, Mianyang China. The bacterium was sub-cultured on LB medium. The bacterial inoculum was produced by growing the pathogen for 48 h on LB medium at 27 °C. Sterile distilled water (SDW) was poured on LB plates containing bacterial growth. With the help of sterile cotton swabs, the bacterial growth was scrapped off the surface of plates and re-suspended in 0.85% saline (salt) solution [49]. Using spectrophotometer, the suspension was adjusted to 10^8^ cfu/mL (OD_600_ = 0.3) [50] and then used for the in vitro and in planta experiments.

### 4.2. Collection of Plant and Extract Preparation

Plants of *Conyza canadensis* were collected from the sub-mountainous dry-lands of Sichuan Province, P.R. China. The plants were authenticated by a botanist (School of Life Science and Engineering, Southwest University of Science and Technology, China). The green top part of *C. canadensis* was dried in shade (for 2 weeks) after watery washed, disinfected, and rinsed with sterilized distilled water. The dried plant material was then grounded to make a fine powder that can pass through 100 mm sieve. The dried fine powder was soaked in ethanol (50 g/200 mL) and kept for stirring for 48 h. After 48 h it was filtered through double layers of muslin, centrifuged for 10 min at 9000 rpm and filtered again through Whatman filter study No. 41 to obtain a clear filtrate. Using rotatory vacuum evaporator, the filtrate was evaporated and dried at 40 °C under reduced pressure. The extracted yield stored in a small bottle in fridge at 4 °C and checked for in vitro anti-bacterial activity in different concentrations.

### 4.3. Evaluation of Anti-Bacterial Activity

The antibacterial activity of ethanol extract of *C. canadensis* was examined against *R. solanacearum* by agar well diffusion [51]. The dried extract of *C. canadensis* was dissolved in methanol to get the concentration of 50, 100, 150, 200, 250 and 300 mg mL^−1^ and used for antibacterial activity. At first, 25 mL LB medium containing 0.5 mL bacterial suspension (10^8^ cfu) was poured in each plate and allowed to cool. Using sterilized 2 mm diameter borer, total of 8 wells were punched in each plate. Six wells each was poured with 10 µL extract with increasing concentration, one well with 10 µL of methanol (negative control) and one with 10 µL of ampicillin @ 4 mg mL^−1^ (positive control). The plates were incubated at 28 °C for 24 h. Antibacterial activity was estimated by the size (diameter in mm) of growth inhibition zones [52]. Completely randomized design (CRD) with five replications was used. The experiment was repeated once. Data of the two experiments were compared using t-test and pooled together after finding no significant difference between the treatments of both experiments.

### 4.4. SEM Studies

The bacterial cells after treated with *C. canadensis* extracts were subjected to SEM analysis for the observations of morphologic alterations. The method for preparing the bacterial cells for SEM analysis was used as described by [36]. Briefly, from the relevant inhibition zones (treated and control) small agar pieces were cut out and fixed in 2.5% glutaraldehyde solution for one hour at 4 °C. Next, these pieces were fixed in 1% osmium tetroxide for two hours after washing with phosphate buffer. This was followed by washing for ten minutes in phosphate buffer and dehydration with the help of graded ethanol. Fully dried pieces coated with gold sputtering were subjected to SEM analysis.

### 4.5. Green House Experiment

The *C. canadensis* plants were shade dried for two weeks, grounded into fine powder and used for in planta antibacterial evaluation against bacterial wilt disease in tomato under greenhouse conditions.

#### 4.5.1. Experiment 1: Application Time and Dose Effect

To find out the influence of application time of dried *C. canadensis* powder, four different doses (0, 8, 16 and 24 g) were applied separately in three different timings viz. 0 days before transplanting (DBT), 8 DBT and 16 DBT. Briefly, plastic pots of 15 cm diameter each were filled with 1 kg steam-sterilized soil. Four different doses i.e., 0 g, 8 g, 16 g and 24 g were mixed with 1 kg steam-sterilized potted soil at three different application times 0, 8 and 16 DBT. After mixing the dried powder with sterilized potted soil, each pot was then inoculated with 20 mL of the bacterial suspension (OD_600_ = 0.3 corresponding to 108 cfu/mL). The bacterial inoculum was poured at the center of the pot [50]. After inoculating the soil, healthy and uniform size tomato seedlings were transplanted to these pots (1 seedling/pot). Plants were watered and fertilized according to horticultural recommendations. Completely randomized design with two factorial (application times and doses) arrangement was used. Each treatment was replicated 4 times. The data on different parameters were taken 60 days post-transplanting. The experiment was repeated once. Data of the two experiments were compared using t-test and pooled together after finding no significant difference between the treatments of both experiments.

#### 4.5.2. Experiment 2: Plastic Mulching Duration and Dose Effect

To find out the influence of plastic mulching duration and different doses of dried *C. canadensis* powder, four different doses were applied separately with different plastic mulching duration i.e., 0, 5 and 10 days. Briefly, plastic pots of 15 cm diameter each was filled with 1 kg steam-sterilized soil. Four different doses i.e., 0 g, 8 g, 16 g and 24 g were mixed separately with 1 kg steam-sterilized potted soil in three groups of pots. After mixing the dried powder with sterilized potted soil, each pot was then inoculated with 20 mL of the bacterial suspension (OD_600_ = 0.3 corresponding to 10^8^ cfu/mL). One group of pot was plastic-mulched for 5 days, one for 10 days and one for 0 day (without mulching) and transplanted with healthy and uniform size tomato seedlings (1 seedling/pot). Remaining experimental procedure was same as above for experiment 1. Polyethylene plastic bought from local market was used for mulching and applied to cover the soil surface.

### 4.6. Data Parameters and Statistical Analysis

The experiment was brought to end after 60 days of transplantation. Data were recorded on different parameters such as (i) severity of the disease using AUDPC, (ii) length of shoot (cm), (iii) length of root (cm), (iv) fresh bio-mass of plant (g) and (vi) bacterial population cfu/g soil. For shoot and root lengths data, main shoot and root of each plant were measured using clear plastic ruler and for fresh biomass data, weight of the entire plant was taken using electronic balance. For AUDPC and bacterial population the following procedure was used.

### 4.7. Area Under Disease Progressive Curve (AUDPC)

A particular scale of wilting of plant was used for the measurement of disease severity. Different scale categories indicated different stages of plant wilting such as: 1 = no symptoms, 2 = less than half of the foliage wilted, 3 = half of the foliage being wilted; 4 = all of the foliage being wilted; 5 = whole plant wilted and dead. Percent disease severity was calculated for each treatment by using the formula [53].

$$\mathrm{DSI}\%=\frac{\sum n}{5N}\times 100$$

where, ∑n = summation of ratings of all plants scored i.e., ∑ (1A+2B+3C+4D+5E) where A, B, C, D and E are the number of the plants of categories 1, 2, 3, 4 and 5 respectively; N = the total number of plants used, and 5 = biggest category of the scale. The percent disease severity data taken at two-week interval were converted to AUDPC values [54].

$$\mathrm{ADPC}=\sum_{i=0}^{n}\left(\frac{X_{i+1}+X_{i}}{2}\right)(T_{i+1}-T_{i})$$

where, n = Total number of assessments, T_i_ = Time at ith assessment and X_i_: Infection expressed in quantity at the ith assessment.

### 4.8. Population Dynamics of R. solanacearum

To know the changes in bacterial population in the soil under the influence of different treatments, three soil cores were taken using 10 mm diameter cork borer from the root’s vicinity at the depth of 12 cm per replicate. All the soil cores from all the replicates of the same treatment were mixed to get a composite sample [55,56]. Three subsamples from the composites sample was taken and serially diluted up to 10^−7^ separately. Suspension of 100 μL each from three serially diluted 10^−7^ subsamples were poured on TZCNA selective medium plate separately [57]. After incubating the plates at 28 °C for 48 h, off-white bacterial colonies with a red center were counted and cfu/g of soil was calculated.

### 4.9. Statistical Analysis

All the data were presented as mean ± standard deviation of six replicates. Statistical analysis was done by means of IBM SPSS Statistics for Windows, Version 20.0. (Armonk, NY: IBM Corp.). In vitro data were analyzed by applying CRD while data from greenhouse experiments were analyzed by applying CRD with two factorial arrangements. Treatment means were compared using Fisher’s Protected LSD test and separated by lower case lettering [58].

## 5. Conclusions

Soil amendment through dried powder of *Conyza canadensis* clearly reduced the soil population of *Ralstonia solanacearum*, caused reduction in disease severity and enhanced plant growth parameters. In vitro evaluation of *C. canadensis* extract and morphologic observation of treated bacterial cells also confirmed its direct antibacterial effect against *R. solanacearum*. To sum up, our findings suggest that the use of dried powder of *C. canadensis,* by limiting chemical use, could act as low-cost and effective disease management measure for BW in tomato grown and possibly other crops.
